# Irritable Bowel Syndrome in Dialysis Patients and Symptom Check List Revised (SCL 90-R) Screening

**DOI:** 10.5152/eurasianjmed.2021.20412

**Published:** 2021-10

**Authors:** Ali Yılmaz, Pınar Gökçen, Hatice Yılmaz, Can Hüzmeli, Abdülkerim Yılmaz

**Affiliations:** 1 Department of Internal Medicine, Cumhuriyet University School of Medicine, Sivas, Turkey; 2 Department of Gastroenterology, Cumhuriyet University School of Medicine, Sivas, Turkey; 3 Department of Internal Medicine, Tire State Hospital, İzmir, Turkey; 4 Department of Nephrology, Cumhuriyet University School of Medicine, Sivas, Turkey

**Keywords:** Dialysis patients, irritable bowel syndrome, symptom check list revised

## Abstract

**Objective:**

Irritable bowel syndrome (IBS) is a frequently seen functional bowel disease. Although not life-threatening, it impairs quality of life and leads to economic losses. IBS symptoms are widespread in dialysis patients. Psychopathological disorders are known to increase in both IBS and dialysis patients. The purpose of this study was to investigate the prevalence of IBS, IBS-related factors, and psychopathological disorders in patients.

**Materials and Methods:**

One hundred fifty patients followed-up in hemodialysis (HD) or peritoneal dialysis (PD) programs were included in this prospective study. Patients were divided into groups with and without diagnoses of IBS based on the Rome-III diagnostic criteria. The Symptom Check List Revised (SCL90-R) test was then applied to the patients. Patients with and without IBS were compared according to the scores obtained from the questionnaire.

**Results:**

IBS was determined in 59 (39.3%) of the dialysis patients. The prevalence of IBS was significantly higher in women (*P =* .030). The presence of coronary artery disease (CAD) and use of erythropoietin (EPO) were significantly higher in patients with IBS (*P* = .029, *P* = .031). Somatization, obsessive-compulsive disorder, interpersonal sensitivity, depression, anxiety, phobic anxiety, psychoticism, and additional items were also higher in patients with IBS. Subscale scores for somatization, depression, and additional parameters in dialysis patients with IBS were above the threshold values for screening.

**Conclusion:**

IBS is common in dialysis patients. The presence of CAD or use of EPO were frequently observed in dialysis patients with IBS, and psychopathologies in depression, somatization, and additional sub-parameters were also higher in these patients.

## Introduction

Irritable bowel syndrome (IBS) is a functional bowel disease characterized by chronic and recurrent abdominal pain, diarrhea, constipation, or abdominal distension. The worldwide prevalence is approximately 10–15%.^1-^^3^ No definite organic cause of IBS has yet been identified, although factors such as impaired motility, visceral hypersensitivity, inflammation, neurotransmitter imbalance, and stress have been implicated in the pathogenesis.^1^ The Rome III criteria, the updated form of the IBS diagnostic criteria, were set out at the Los Angeles Digestive Diseases Meeting.^4^ The reported sensitivity and specificity of the Rome III criteria are 70.7% and 87.8%, respectively.^5^

Symptoms in IBS patients are sometimes exacerbated by stress and may be related to psychiatric diseases.^6^ Psychiatric comorbidities are seen in 42–61% of IBS patients. Studies have shown 2-, 3-, and 6-fold higher incidences of anxiety, depression, and hypochondriasis, respectively, in IBS patients.^7^ The Symptom Check List Revised (SCL90-R) is a psychological screening list that evaluates the severity of mental symptoms and their related domains. SCL90 consists of 10 subgroups including somatization, obsessive compulsive, interpersonal sensitivity, depression, anxiety, anger, phobic anxiety, paranoid ideation, psychosis, and additional conditions.^8^

Chronic kidney disease (CKD) is associated with depression and anxiety and is known to impair quality of life in hemodialysis (HD) patients compared to the normal population.^9,10^ The incidence of gastrointestinal system (GIS) symptoms is higher in HD patients. Among these GIS symptoms, IBS findings are more common than in the general population and occur in 11–44% of HD patients.^11,12^ Panic disorder and anxiety have been shown to be associated with IBS.^13^ Major depression and anxiety are also known to be more common in patients with CKD.^14^ The aim of this study was to determine the prevalence of IBS and the factors associated with IBS and to investigate the psychological symptoms of patients who received HD or peritoneal dialysis (PD) with indications of CKD.

## Materials and Methods

One hundred fifty volunteer patients followed-up in the HD or PD programs in our center between November 2014 and June 2015 were prospectively included in this study. Patients with fecal occult blood positivity together with weight loss, with a family or personal history of colon cancer or antibiotic use within the previous 1 month, with known gastrointestinal diseases such as Crohn’s disease, ulcerative colitis, and celiac disease, were excluded. Patients with pathological findings at endoscopy and colonoscopy, which were performed on all patients in the previous last year, were also excluded. Patients with evidence of angina pectoris, myocardial infarction, or coronary revascularization procedures within the previous 6 months and exhibiting any of the alarm symptoms (weight loss, family history of cancer, or hematochezia) and findings detected at differential diagnosis of IBS were also excluded from the study. Patients who had used GIS motility regulator drugs were also not included in the study. Finally, patients with previously diagnosed psychological disorders were also excluded.

Sociodemographic characteristics such as age, gender, marital status, educational status, income level, smoking status, and duration of dialysis and clinical laboratory data were recorded. Patients’ incomes were classified as low (<$400), modiserate (≥$400–800), or high (>$800). The applicable criteria for the diagnosis of IBS at the time when ethical approval was obtained from the local ethics committee of Cumhuriyet University School of Medicine (number 2014-10/01 dated 23.10.2014) and when the study was performed were the Rome-III criteria. These criteria were therefore employed for the diagnosis of IBS in this study. According to these criteria, diagnosis of IBS is based on the presence of at least two of (1) symptoms being relieved with defecation, (2) symptoms manifesting with frequency of bowel movements, and (3) changes in the consistency of stool, in addition to symptoms of recurrent abdominal pain or discomfort, starting at least 6 months before diagnosis and observed for at least 3 days every month within the previous 3 months.^4^

SCL90-R was applied to all participants enrolled in the study. The patients scored the 90 questions on the scale according to the severity of their symptoms within the previous 3 months—no problem (0), mild (1), moderate (2), quite disturbing (3), and extremely disturbing.^4^ Three different general scores were calculated from the SCL90-R scale: general symptom level (GSL), positive symptom total (PST), and positive symptom level (PSL). The GSL was estimated by summing the scores obtained from the responses to all the questions and dividing this by the number of questions. Scores greater than one indicated the presence of a psychopathological condition. PST represents the sum of other items, excluding scores assigned to the “no choice item”. PSL was estimated by dividing the sum of scores of all items with PST excluding those assigned to “no choice”.^8^ Calculations were performed for GSL and somatization, obsessive-compulsive symptoms, interpersonal sensitivity, depression, anxiety, anger, phobic anxiety, paranoid ideation, psychoticism, and subgroups of additional items for all patients. Based on the scores obtained, values above one were considered significant, and the individual concerned was considered to have a disposition to psychopathological symptoms.

### Statistical Analysis

The results obtained from our study were loaded onto SPSS version 22.0 (IBM SPSS Corp.; Armonk, NY, USA) software. If parametric test assumptions were fulfilled (Kolmogorov–Smirnov) in the evaluation of the data, the significance test was used to compare the difference between 2 means. When parametric test assumptions were not met, the Mann–Whitney *U* test and Chi-square test were applied. *P* < .05 was considered statistically significant.

## Results

Of the 150 patients included in the study, 80 (53.3%) were male and 70 (46.7%) were female. Fifteen (10%) patients were aged 18–29 years, 11 (7.3%) were aged 30–41 years, 17 (11.3%) were aged 42–53 years, 44 (29.3%) were aged 54–65 years, and 63 (42%) were aged >65. The mean age of the patients was 59.07 ± 16.76 years. The HD and PD programs included 142 (94.6%) and 8 (5.4%) cases, respectively. One hundred fifteen (76.7%) patients had been attending dialysis programs for 0–5 years, 28 (18.7%) for 6–11 years, 4 (2.7%) for 12–17 years, 2 (1.3%) for 18–23 years, and 1 (0.6%) for 24–29 years. Five (3.3%), 72 (48%), 60 (40%), and 105 (70%) patients had histories of the clinical conditions renal transplantation (tx), diabetes mellitus (DM), CAD, and hypertension (HT), respectively. Fifty-three patients (35.3%) were using EPO and 74 (49.3%) were using antiphosphate drugs, while 97 (64.7%) patients were not using EPO and 76 (50.7%) were not using antiphosphate drugs (Table 1).

Thirty-four (39.3%) female and 25 (42.4%) male were diagnosed with IBS. The incidence of IBS was significantly higher in female gender (*P* = .03). Body mass index (BMI) values did not differ significantly among patients with IBS (*P* = .124). IBS was diagnosed in 56 (39.4%) of the HD and 3 (37.5%) of the PD patients, but no significant difference was determined between the types of dialysis in terms of prevalence of IBS (*P* = .913). No significant difference was also observed in the duration of dialysis between patients with and without IBS (*P* = .370). When patients with IBS were analyzed in terms of clinical characteristics, histories of renal tx, DM, CAD, or HT were present in 2 (3.4%), 33 (55.9%), 30 (50.8%), and 45 (76.3%) patients, respectively (Table 2). IBS was significantly more frequently seen among these patients in those with CAD compared to those without (*P* = .029). Among the patients with IBS, 27 (45.8%) were using EPO and 34 (57.6%) were using antiphosphate drugs, while 32 (54.2%) were not using EPO and 25 (42.4%) were not using antiphosphate drugs. Use of EPO constituted a significant risk factor for IBS but not antiphosphate use (*P* = .031 and 0.102, respectively).

No significant difference in albumin, C-reactive protein, blood urea nitrogen, creatinine, calcium (Ca), phosphorus, alkaline phosphatase, aspartate aminotransferase, alanine aminotransferase, iron, serum iron binding capacity, ferritin, hemoglobin, dialysis adequacy dose (Kt/V), potassium (K+), pre-HD K+, post-HD K+, and parathormone (PTH) values was observed between patients with IBS and those without (*P* = 0.077, 0.516, 0.053, 0.085, 0.148, 0.067, 0.411, 0.711, 0.691, 0.829, 0.192, 0.841, 0.819, 0.373, 0.955, 0.242, 0.992, and 0.602, respectively) (Table 3).

In terms of SCL90-R scale scores, the overall symptom score was higher in patients with IBS than in non-IBS patients (*P* = .001). Subscale parameter scores for somatization, obsessive-compulsive disorder, interpersonal sensitivity, depression, anxiety, phobic anxiety, psychoticism, and additional items were higher in patients with IBS (*P* = .001, .001, .026, .001, .001, .001, .001, and .001, respectively). No significant difference was determined in terms of the parameters of anger and paranoid ideation based on the presence or absence of IBS (*P* = .051 and .734, respectively). IBS patients’ somatization, depression, and additive subscale scores were higher than one, the threshold value for screening. In terms of the other parameters, although the scores were not higher than one, all scores were higher in individuals with IBS than in those without (Table 4).

## Discussion

IBS is a frequently seen functional disorder that disrupts quality of life and leads to serious economic losses.^11^ GIS-related symptoms are common in CKD; among them, IBS symptoms being particularly frequently seen.^11,12^ Symptoms in patients with IBS may be triggered by stress and may be related to psychiatric disorders. Anxiety, depression, somatoform, and phobic disorders are frequently seen comorbidities.^15,16^ The present study demonstrates, for the first time in the literature, that the prevalence of IBS in HD or PD patients is high and related to the presence of CAD and EPO use. Additionally, and also for the first time in the literature, the SCL90-R results showed that the dialysis patients were predisposed to somatization, depression and additional subparameters, obsessive-compulsive, interpersonal sensitivity, anxiety, phobic anxiety, and psychotic disorder.

Previous studies have reported IBS symptoms in approximately 20% of women and 10% of men.^17^ In one study of HD and PD patients, 60.3% of patients diagnosed with IBS were female and 39.7% were male.^12^ In the present study, the prevalence of IBS was significantly higher in the women, in accordance with studies performed in the community in general and among dialysis patients (*P* = 0.03). The reasons for the higher incidence rates of IBS in female gender are that women present to hospital more frequently than men, that women are more sensitive to psychological stress conditions, that female hormones affect visceral sensitivity and lower the pain threshold, and differences in serotonin levels in the central nervous system.^18^ Drossman et al. [1] reported that the prevalence of IBS decreased with age. In a study of 5009 individuals in the United States, 67.3% of cases were aged 25–54 years.^19^ In a study of 236 patients undergoing HD treatment, the mean ages of patients with and without IBS were 53.1 ± 13.9 and 50.7 ± 15.4 years, respectively. Andrews et al. [20] reported a higher prevalence of IBS in individuals with lower education and income levels. However, another study of dialysis patients reported that level of education was not significant for IBS.^21^ A different study reported an inverse relationship between socioeconomic status and the prevalence of IBS in individuals not receiving dialysis.^22^ In the present study, although education and income status were not related to diagnosis of IBS, the discrepancy with previous findings may derive from relative responses and the diagnostic criteria used. Fiderkiewicz et al. [23] investigated factors associated with IBS symptoms in HD patients and observed no significant correlation between BMI and IBS, consistent with the present study.

Afsar et al. [21] reported that 29.2% of 236 dialysis patients were diagnosed with IBS according to the Rome II diagnostic criteria. In another study of 128 dialysis patients, 44.5% of patients had IBS, but the type of dialysis did not create a significant difference in terms of the prevalence of IBS.^12^ The prevalence of IBS in the present study was 39.3%, but type of dialysis and number of sessions were not significant for IBS. We think that the variation in prevalence rates between the present research and other studies is due to differences in the diagnostic criteria used. One study reported a higher prevalence of IBS in unmarried patients compared with married patients (7.7% vs 5.9%).^20^ In another study of HD patients, 40% of married and 45.2% of unmarried patients had IBS, but this difference was not significant [21]. Kim et al. [24] reported no significant relationship between prevalence of IBS and living alone. In the present study, marital status was not significant for the diagnosis of IBS. A study conducted in the United Kingdom reported a strong correlation between smoking and prevalence of IBS, while no such correlation was found in other studies.^25,26^ Similarly in the present study, no significant relationship was found between IBS and smoking. In addition, we determined no difference between the groups in terms of presence or absence of sleep disorders in patients with IBS. Although Rotem et al. [27] found significantly higher rates of sleep disturbances in 18 patients with IBS, we think that differences between the various studies may be due to the subjective responses given by the patients.

In terms of clinical features, we observed no significant association between the prevalence of IBS in dialysis patients and presence of renal tx, DM, HT, or antiphosphate use. However, a significant difference was determined in the prevalence of IBS among CAD patients and EPO users. These results are consistent with those of Afsar et al. [21] in the context of correlations between the prevalence of IBS and the presence of renal tx, DM, and HT and use of antiphosphates being insignificant. However, our results differ from theirs concerning the presence of CAD or EPO use. In another study involving HD patients, Gök et al. [28] demonstrated that the presence of CAD, HT, and DM created no significant difference in terms of IBS symptoms. The reason for the greater prevalence of CAD in IBS patients remains unclear, although studies have suggested that IBS patients are more vulnerable to psychosocial stress and undergo more medical consultations for GIS symptoms or physical comorbidities. In addition, the underlying mechanism in patients with CAD may be a hypoxic environment due to circulatory disorder.^29,30^ Regular intravenous infusion may increase emotional stress in patients using EPO, and this may represent a triggering mechanism. The findings of the present study confirm the significance of the presence of CAD or EPO use in the context for the prevalence of IBS, but we think this should now be corroborated by further studies. A study of 196 patients in the HD program reported a greater number of IBS symptoms in post-dialysis hypopotasemic patients compared with normopotasemic patients.^23^ In the present study, neither K+ levels nor the other laboratory parameters were significant for IBS. Similar results were obtained in a study performed among HD patients in which no significant association was found between laboratory findings and IBS.^21^ In another study including 80 HD and 80 healthy controls, PTH, Kt/V, and Ca were not significantly correlated with IBS symptoms.^28^

Studies investigating psychiatric disorders in patients with IBS have reported significant prevalences of major depression (33%), generalized anxiety disorder (40%), and panic disorder (25–50%). It has been suggested that anxiety is associated with IBS in the short term, but with depression in chronic patients.^7^ Garakani et al. [31] also found a strong correlation between anxiety disorders and IBS. Cole et al. [32] reported that 12.8% of IBS patients were diagnosed with depression, while depression was present in 6% of individuals without IBS. Another study reported higher Hamilton Anxiety and Depression Scale scores in patients with IBS compared to a control group.^33^ Similarly, a study conducted using the Short Form 36 (SF-36) Quality of Life Scale reported significantly lower scores for all subscales except for physical subscores.^34^ In the present study, patients with and without IBS were compared using SCL90-R scale scores. Accordingly, overall symptom scores and scores related to somatization, obsessive-compulsive disorder, interpersonal sensitivity, depression, anxiety, phobic anxiety, psychoticism, and sub-parameters of additional items were significantly higher in patients with IBS. The difference was not significant in the anger and paranoid ideation sub-parameters. The somatization, depression and additional item (sleep disorder, poor appetite, and feelings of guilt) subscale scores of IBS patients were higher than the screening threshold value. In terms of other parameters, patients with IBS always registered higher scores than patients without IBS, although these did not exceed the threshold value for screening. We encountered no previous studies using SCL90-R test in dialysis patients with IBS. A study investigating the factors associated with IBS in patients with dialysis employed the Kidney Disease Quality of Life Cognitive Function Scale (KDQOL-CF) and reported significantly lower quality of life scores in patients with IBS. However, IBS was not associated with anxiety or depression.^23^ Another study involving dialysis patients and using the Hospital Anxiety and Depression Scale reported a greater number of psychological disorders in patients with IBS. In addition, higher incidences of anxiety, depression, or coexistence of the two were determined in IBS patients entering dialysis compared to non-IBS patients.^12^ Finally, another study determined higher Beck Depression Index scores and lower SF-36 Quality of Life Scale scores in dialysis patients with IBS.^21^

There are a number of limitations to the present study. In particular, due to its single-center nature, the number of patients included was limited. Another important limitation was the absence of a healthy control group.

In conclusion, the prevalence of IBS in dialysis patients is high, and the presence of CAD or use of EPO may be associated with IBS. Furthermore, dialysis patients with IBS have a strong tendency to manifest general symptoms, mainly in the parameters of somatization, depression, and subgroups of additional items (sleep disorder, loss of appetite, and feelings of guilt), as well as obsessive-compulsive disorder, interpersonal sensitivity, anxiety, phobic anxiety, and psychotic disorder. The potential presence of psychopathological comorbidities, mainly depression and somatization, should be taken into consideration when planning the management of IBS in dialysis patients for improved therapeutic success.

**Main Points**

- Irritable bowel syndrome (IBS) is a common functional disease of the intestinal tract. It is known that gastrointestinal symptoms are more common in dialysis patients.
- Also, psychopathological disorders are closely related to IBS and dialysis patients. Increasing psychopathological disorders in dialysis patients with IBS may decrease their quality of life.
- Considering psychopathological comorbidities in the management of dialysis patients with IBS may increase treatment success.

## Figures and Tables

**Table 1.** Patients’ Sociodemographic and Clinical Characteristics

	Total n, (%)
Gender	
Male	80 (53.3)
Female	70 (46.7)
Marital status	
Married	134 (89.3)
Single	16 (10.7)
Smoker	
Present	32 (21.3)
Absent	118 (78.7)
Sleep disorders	
Present	84 (56)
Absent	66 (44)
Renal transplantation	
Present	5 (3.3)
Absent	145 (96.7)
DM	
Present	72 (48)
Absent	78 (52)
CAD	
Present	60 (40)
Absent	90 (60)
HT	
Present	105 (70)
Absent	45 (30)
EPO	
Present	53 (35.3)
Absent	97 (64.7)
Age (years)	
18–29	15 (10.0)
30–41	11 (7.3)
42–53	17 (11.3)
54–65	44 (29.3)
>65	63 (42.0)
Education level	
Illiterate	31 (20.7)
Primary	57 (38)
Secondary	21 (14)
High School	25 (16.6)
University	16 (10.7)
Income level	
Low	72 (48.0)
Moderate	74 (49.3)
High	4 (2.7)
BMI (kg/m^2^)	
<18.5	6 (4.0)
18.5–24.9	60 (40.0)
25–29.9	56 (37.3)
30–34.9	19 (12.7)
35–39.9	5 (3.3)
≥40	4 (2.7)

BMI: body mass index, CAD: coronary artery disease, DM: diabetes mellitus, EPO: erythropoietin, HT: hypertension

**Table 2.** Patients’ Sociodemographic and Clinical Characteristics according to Presence of IBS

	IBS (+) n, (%)	IBS (–) n, (%)	Total n, (%)	Result
Gender				
Female	34 (57.6)	36 (39.6)	70 (46.7)	*P* = .030*
Male	25 (42.4)	55 (60.4)	80 (53.3)	
Marital status				
Married	53 (89.8)	81 (89)	134 (89.3)	*P* = .874
Single	6 (10.2)	10 (11)	16 (10.7)	
Smoker				
Present	14 (23.7)	18 (19.8)	32 (21.3)	*P* = .564
Absent	45 (76.3)	73 (80.2)	118 (78.7)	
Sleep disorders				
Present	33 (55.9)	51 (56)	84 (56)	*P* = 1.000
Absent	26 (44.1)	40 (44)	66 (44)	
Renal transplantation				
Present	2 (3.4)	3 (3.3)	5 (3.3)	*P* = .975
Absent	57 (96.6)	88 (96.7)	145 (96.7)	
DM				
Present	33 (55.9)	39 (42.9)	72 (48)	*P* = .117
Absent	26 (44.1)	52 (57.1)	78 (52)	
CAD				
Present	30 (50.8)	30 (33)	60 (40)	P = .029*
Absent	29 (49.2)	61 (67)	90 (60)	
HT				
Present	45 (76.3)	60 (65.9)	105 (70)	*P* = .177
Absent	14 (23.7)	31 (34.1)	45 (30)	
EPO				
Present	27 (45.8)	26 (28.6)	53 (35.3)	*P* = .031*
Absent	32 (54.2)	65 (71.4)	97 (64.7)	
Age (years)				
18–29	7 (11.9)	8 (8.8)	15 (10.0)	*P* = .830
30–41	3 (5.1)	8 (8.8)	11 (7.3)	
42–53	8 (13.6)	9 (9.9)	17 (11.3)	
54–65	17 (28.8)	27 (29.7)	44 (29.3)	
>65	24 (40.7)	39 (42.9)	63 (42.0)	
Education level				
Illiterate	16 (27.1)	15 (16.5)	31 (20.7)	*P* = .469
Primary	23 (39.0)	34 (37.4)	57 (38)	
Secondary	6 (10.2)	15 (16.5)	21 (14)	
High School	9 (15.3)	16 (17.6)	25 (16.6)	
University	5 (8.5)	11 (12.1)	16 (10.7)	
Income level				
Low	34 (57.6)	38 (41.8)	72 (48.0)	*P* = .157
Moderate	24 (40.7)	50 (54.9)	74 (49.3)	
High	1 (1.7)	3 (3.3)	4 (2.7)	
BMI (kg/m²)				
<18.5	0 (0.0)	6 (6.6)	6 (4.0)	*P* = .124
18.5–24.9	25 (42.4)	35 (38.5)	60 (40.0)	
25–29.9	20 (33.9)	36 (39.6)	56 (37.3)	
30–34.9	10 (16.9)	9 (9.9)	19 (12.7)	
35–39.9	1 (1.7)	4 (4.4)	5 (3.3)	
≥40	3 (5.1)	1 (1.1)	4 (2.7)	

* *P* < .05 significant.

BMI: body mass index, CAD: coronary artery disease, DM: diabetes mellitus, EPO: erythropoietin, HT: hypertension

**Table 3.** Comparison of Laboratory Findings of Patients with and without IBS

	IBS (+) X¯ ± S	IBS (–) X¯ ± S	Result
Albumin	3.66 ± 0.56	3.48 ± 0.64	*P* = .077
CRP	42.90 ± 63.59	29.75 ± 40.37	*P* = .516
BUN	41.72 ± 19.74	49.76 ± 21.46	*P* = .053
Creatinine	4.88 ± 2.36	5.77 ± 2.87	*P* = .085
Ca	8.47 ± 0.79	8.29 ± 0.87	*P* = .148
P	4.30 ± 1.52	4.72 ± 1.25	*P* = .067
ALP	133.08 ± 83.22	130.79 ± 99.41	*P* = .411
AST	23.91 ± 43.92	24.26 ± 45.10	*P* = .711
ALT	22.15 ± 42.64	18.30 ± 20.25	*P* = .691
Iron	63.49 ± 37.83	62.06 ± 35.91	*P* = .829
SIBC	218.68 ± 57.27	206.89 ± 51.45	*P* = .192
Ferritin	331.32 ± 329.56	318.94 ± 392.66	*P* = .841
Hb	10.73 ± 1.74	10.80 ± 1.72	*P* = .819
Kt/V	1.27 ± 0.28	1.22 ± 0.31	*P* = .373
Pre HD K +	4.81 ± 0.74	4.80 ± 0.79	*P* = .955
Post HD K +	3.64 ± 0.55	3.66 ± 0.52	*P* = .242
K	4.40 ± 0.87	4.41 ± 0.78	*P* = .992
PTH	331.32 ± 329.56	318.94 ± 392.66	*P* = .602

* *P* < .05 significant.

ALP: alkaline phosphatase, ALT: alanine aminotransferase, AST: aspartate aminotransferase, BUN: blood urea nitrogen, Ca: calcium, CRP: C-reactive protein, Hb: hemoglobin, Kt/V: (K, dialyzer clearance of urea; t, dialysis time; V, volume of distrubition of urea), K: potassium, P: phosphorus, PTH: parathormone, SIBC: serum iron binding capacity

**Table 4.** Comparison of Patients with and without IBS According to Symptom Check List Revised (SCL90-R) Scale Scores

	IBS (+) X¯ ± S	IBS (–) X¯ ± S	Result
Somatization	1.56 ± 0.85	0.94 ± 0.78	***P* = .001** *
Obsessive-compulsive	0.96 ± 0.72	0.60 ± 0.57	***P* = .001** *
Interpersonal sensitivity	0.57 ± 0.56	0.40 ± 0.47	***P* = .026** *
Depression	1.04 ± 0.71	0.67 ± 0.59	***P*= .001** *
Anxiety	0.98 ± 0.62	0.56 ± 0.50	***P* = .001** *
Anger	0.76 ± 0.68	0.58 ± 0.66	*P* = .051
Phobic anxiety	0.70 ± 0.69	0.32 ± 0.47	***P* = .001** *
Paranoid ideation	0.33 ± 0.36	0.34 ± 0.43	*P* = .734
Psychoticism	0.45 ± 0.36	0.27 ± 0.34	***P* = .001** *
Additional items	1.47 ± 0.81	0.93 ± 0.72	***P* = .001** *
Overall symptom score	0.92 ± 0.47	0.58 ± 0.45	***P* = .001** *

* *P* < .05 significant.
